# Identification of human remains using Rapid DNA analysis

**DOI:** 10.1007/s00414-019-02186-y

**Published:** 2019-11-28

**Authors:** Rosemary S. Turingan, Jessi Brown, Ludmila Kaplun, Jake Smith, Jenna Watson, Derek A. Boyd, Dawnie Wolfe Steadman, Richard F Selden

**Affiliations:** 1 ANDE Corporation, 266 Second Avenue, Waltham, MA 02451 USA; 2 Department of Anthropology, The University of Tennessee Knoxville, Knoxville, TN 37996 USA

**Keywords:** Disaster victim identification, Rapid DNA identification, Short tandem repeat, FlexPlex

## Abstract

Rapid identification of human remains following mass casualty events is essential to bring closure to family members and friends of the victims. Unfortunately, disaster victim identification, missing persons identification, and forensic casework analysis are often complicated by sample degradation due to exposure to harsh environmental conditions. Following a mass disaster, forensic laboratories may be overwhelmed by the number of dissociated portions that require identification and reassociation or compromised by the event itself. The interval between the disaster and receipt of victim samples at a laboratory is critical in that sample quality deteriorates as the postmortem interval increases. When bodies decompose due to delay in collection, transport, and sample processing, DNA becomes progressively fragmented, adversely impacting identification. We have previously developed a fully automated, field-forward Rapid DNA identification system that produces STR profiles (also referred to as DNA IDs or DNA fingerprints) from buccal and crime scene samples. The system performs all sample processing and data interpretation in less than 2 h. Here, we present results on Rapid DNA identification performed on several tissue types (including buccal, muscle, liver, brain, tooth, and bone) from exposed human bodies placed above ground or stored in a morgue/cooler, two scenarios commonly encountered following mass disasters. We demonstrate that for exposed remains, buccal swabs are the sample of choice for up to 11 days exposure and bone and tooth samples generated excellent DNA IDs for the 1-year duration of the study. For refrigerated remains, all sample types generated excellent DNA IDs for the 3-month testing period.

**Electronic supplementary material:**

The online version of this article (10.1007/s00414-019-02186-y) contains supplementary material, which is available to authorized users.

## Introduction

DNA genotyping in a conventional laboratory has become a standard tool for identification of victims from mass casualty events including the World Trade Center attack [1, 2], natural phenomena [3–5], plane crashes [6, 7], and terrorist-related atrocities [8]. The DNA Commission of the International Society for Forensic Genetics (ISFG) provided forensic DNA laboratories recommended guidelines for sample collection and storage (both ante- and postmortem), DNA purification, and genetic typing, with protocols for data management at the disaster sites issued by Interpol [9]. Furthermore, software and bioinformatics tools have been developed to provide necessary associations after DNA IDs have been generated [10, 11].

Depending upon the nature and scale of the event, human remains may not be recovered from the field immediately and then may be stored for a variable period of time in refrigerated or unrefrigerated conditions. Identifications in mass disaster contexts require a multidisciplinary response, and can be based on dental remains, anthropological evidence, and fingerprints, but DNA is the most common identification modality in many responses [12]. However, few jurisdictions in the USA have in-morgue DNA laboratories that are equipped for the high-throughput requirements of mass fatality events [13, 14]. In these cases, victim samples must be shipped to off-site forensic laboratories, further delaying analysis. Decomposed human remains present a significant identification challenge requiring development of highly sensitive detection approaches [12].

Nuclear DNA is typically preferred for forensic identification because it is extremely accurate and provides biparental kinship information [15]. STR typing of nuclear DNA is the most frequently relied upon technique [10, 16] and can distinguish a large number of individuals at a given time. Historically, DNA testing has been used as a last resort for the identification of human remains because it is time-consuming, often requiring several months to years for analysis, and requires sophisticated laboratory facilities staffed with trained DNA analysts. New technology in Rapid DNA has mitigated the need for lengthy analyses by specialists. The ANDE Rapid DNA Identification system, comprising the ANDE 6C instrument, A- and I-Chips, and Expert System software, received the FBI’s National DNA Index System (NDIS) approval for automated processing of buccal swabs in 2018 [17]. The system utilizes a multiplexed assay, FlexPlex27, that interrogates 27 STR loci simultaneously (23 autosomal, 3 Y-chromosomal, and Amelogenin) [18]. NDIS approval is a critical requirement for implementation of Rapid DNA in police stations as stipulated by the Rapid DNA Act of 2017 (https://www.congress.gov/bill/115th-congress/house-bill/510). DNA IDs generated by ANDE can be used to automatically search the FBI’s DNA database using the Rapid DNA Index System (RDIS). Furthermore, ANDE has been shown to successfully type forensically and DVI-relevant samples, including minute amounts of blood and bone [19]. ANDE played an integral role in the identification of remains from the November 2018 Camp Fire, the deadliest wildfire in California history (https://www.cbsnews.com/news/paradise-lost-inside-california-camp-fire-60-minutes/). For the first time, Rapid DNA identification was utilized to identify the majority of victims within hours to days following recovery. The Rapid DNA IDs generated from these unidentified remains were immediately searched against those generated from buccal swabs of family members. Rapid DNA technology is also being used by military and law enforcement in the USA and around the world to combat crime (https://www.congress.gov/bill/115th-congress/house-bill/510), human trafficking (https://www.washingtonpost.com/immigration/homeland-security-to-test-dna-of-families-at-border-in-cases-of-suspected-fraud/2019/05/01/8e8c042a-6c46-11e9-a66d-a82d3f3d96d5_story.html?utm_term=.b4611915101f), and the rape epidemic (https://kentuckystatepolice.org/hq-4-10-19/).

For the last four decades, the Forensic Anthropology Center (FAC) at the University of Tennessee has operated the Anthropology Research Facility (ARF), or Body Farm, a natural outdoor laboratory to study human decomposition. Individuals from around the USA donate their bodies to the program in order to strengthen forensic science. Each donor provides demographic information, medical history, residence history, childhood socioeconomic status, education, and occupation history, among other information. When a donor arrives at the FAC, samples of the blood, hair, and nails are collected at intake, as well as any other samples required of ongoing research. Donors are then placed at the ARF and the decomposition process is documented through daily photos. Longitudinal sampling can occur throughout decomposition. Here, we present results on Rapid DNA identification performed on several tissue types from exposed human donors placed above ground at the ARF and also stored in the FAC morgue cooler, two scenarios commonly encountered following mass disaster scenarios. The goal of the study was to determine the optimal tissue types capable of generating useful DNA identifications over postmortem time as informed by ease of sample collection and processing.

## Materials and methods

### Sample collection

The Forensic Anthropology Center (FAC) at the University of Tennessee provided deceased human subjects (“donors”) for testing and research using ANDE. The FAC enrolled ten deceased human subjects (seven male and three female). Donor enrollment was open to individuals of any sex, age, or ancestry to represent mass fatality scenarios. Two of the ten donors (BD01 and BD02) had previously been autopsied and were used to provide brain samples. Teeth were collected from seven donors who had molars. Eight of the ten donors (LD01-LD08) provided liver samples. Finally, buccal, muscle, and bone samples were collected from every donor. Table 1 provides the sampling and demographic information for each donor. LD06 was placed in the FAC morgue cooler for 3 months before placement above ground at the ARF. Two of the liver donors (LD07 and LD08) were placed in the morgue cooler for 1 month and then placed above ground after 1-month sampling. Remaining donors were placed above ground after sampling at intake. Buccal samples and blood cards were collected at intake for use as references for STR genotyping.

**Table 1:** Sampling matrix and demographic information for each donor

Don or ID	Sex	Sample/tissue types					
		Brain	Buccal	Muscle	Liver	Bone	Teeth
BD01	M	X	X	X		X	
BD02	M	X	X	X		X	X
LD01	M		X	X	X	X	X
LD02	F		X	X	X	X	
LD03	M		X	X	X	X	X
LD04	M		X	X	X	X	X
LD05	F		X	X	X	X	X
LD06	M		X	X	X	X	X
LD07	F		X	X	X	X	X
LD08	M		X	X	X	X	

Placement at the ARF occurred immediately after intake or after the designated time in the morgue cooler. The ARF consists of an approximately 3-acre parcel of land on a forested bluff above the Tennessee River. Medium to large size animals are kept from entering the ARF by double-layered wooden and chain-link fences, but smaller scavengers, such as raccoons, can be considered a taphonomic factor (apart from local climatic changes) that may alter the rate and pattern of decomposition. Donors were placed as they were enrolled, meaning different start and end points for each subject, and then systematically sampled throughout the postmortem period. Sampling continued for a full year after placement.

Tissue types selected for analysis were those considered to be reasonably accessible and likely to be encountered in mass disaster, law enforcement, and military settings. Table 2 shows the sampling strategy for each sample type. The samples types analyzed were as follows:

- Buccal cells: Buccal swabs and approximately 3 mg samples of buccal tissue were taken until time points at which the buccal lining was no longer patent. Some swabs included maggots that inhabited the oral cavity early in decomposition.
- Brain: To simulate traumatic brain injury and avulsion in DVI or combat scenarios, brain tissue was distributed on a piece of drywall to enable evaluation of brain tissue decomposition outside of the body. The remainder of the brain tissue was returned to the cranial cavity to undergo decomposition inside the body.
- Liver: Approximately 3 mg of wet tissue was obtained from the right lobe of the liver, deep in the parenchyma away from the capsule and the main biliary channels and ducts.
- Skeletal muscle: Approximately 3 mg of deep red quadriceps and biceps tissues were obtained. This sampling is to model conditions in which limbs may be severed and immediate reassociation with the correct torso may not be possible. The sample sites were covered with duct tape to eliminate insect access that could affect decomposition rates.
- Tissue swabs: In addition to analyzing gross tissue, swabs were taken of the skeletal muscle and liver to determine if this technique could be applied in the field as a simpler alternative to excising gross tissue samples.
- Teeth: One premolar or molar was submitted, and the root was separated for analysis.
- Bone: Approximately 500 mg samples of the femur shaft (cortical bone) and distal foot phalanges were sampled.

**Table 2:** Sampling strategy for each tissue type. Collection for a given tissue type was discontinued when samples were no longer accessible due to infestation or severe tissue deterioration

Sample/tissue types	Sample amount (intake/subsequent)	Intake	Placement	Day 1	Day 2	Day 4	Week 1	Week 2	Month 1	Month 3	Month 6	Month 12
Brain (splatter)	200 mg/500 mg	X	X	X	X	X	X	X	X	X	X	X
Brain	200 mg/500 mg	X	X	X	X	X	X	X	X	X	X	X
Buccal fragment/swab	200 mg/500 mg	X	X	X	X	X	X	X	X	X	X	X
Liver fragment/swab	200 mg/500 mg	X	X	X	X	X	X	X	X	X	X	X
Muscle fragment/swab	500 mg	X	X	X	X	X	X	X	X	X	X	X
Tooth	1/2 of one root	X							X	X	X	X
Femur	500 mg	X							X	X	X	X
Foot Phalange	1 phalanx	X							X	X	X	X
Rib	500 mg									X	X	X

### Sample processing for Rapid DNA analysis

Samples were immediately stored at – 20 °C until ready for analysis. Sample types were processed as follows:

Tissue (buccal, muscle, and liver) swabs: Swabs were thawed for 15 min prior to direct analysis in ANDE by insertion into either an A-Chip or an I-Chip.

Blood card: A 1 × 3 mm punch was taken from the blood card (Whatman® FTA®) and transferred into a sterile 2-ml microfuge tube. The punch was macerated in TE-4 buffer to ensure that the biological material is released from the substrate and incubated at 50 °C for 15 min. An ANDE swab was inserted into the tube to collect the solution and analyzed on an I-Chip.

Buccal lining: Samples were laid flat in a petri dish while thawing on ice. Samples were either (1) cut into a small fragment (~ 100–150mg) and placed into the bottom of the chip swab chamber or (2) swabbed until the entire sample collection cotton head was saturated with moisture and/or adhered thin tissue fragments followed by insertion into the swab chamber.

Muscle and brain fragments: Samples were laid flat in a petri dish while thawing on ice. Samples were either (1) cut into a small fragment (~ 100mg–150mg) and placed into the bottom of the chip swab chamber or (2) swabbed until the entire sample collection cotton head was saturated with moisture and/or adhered tissue followed by insertion into the swab chamber. Alternatively, a 5–10-mg sample fragment was also collected on a swab and placed into the swab chamber.

Liver fragments: Samples were laid flat in a petri dish while thawing on ice. Samples were either (1) cut into a small fragment (~ 20–30mg) and placed into the bottom of the chip swab chamber or (2) swabbed until the lower third of the cotton head was stained with blood/tissue followed by insertion into the swab chamber. Alternatively, approximately 0.10–0.25 mg sample fragment was collected on a swab and placed into the swab chamber.

Bone: For fresh bone, each drilled bone fragment was cleaned with running water and lightly brushed to remove surface dirt. The bone was immersed in 10% bleach solution in a conical tube and inverted 15–20 times. Bone was transferred into a clean specimen container with sterile water and inverted for an additional 15–20 times. This water rinsing was performed twice. Finally, the bone piece was placed in another specimen tube containing ethanol and inverted 15–20 times prior to letting the bone dry. The cleaned bone was then pummeled into smaller fragments (ranging from dust-like consistency to thin shards of ~ 1/8″ or less) using a hammer and/or a mortar and pestle. Approximately 5–10mg of the prepared bone was placed in a sterile 2-ml microfuge tube. To this, 120 μl of ANDE™ Bone Solution (a commercially available demineralization solution) was added, the tube was vortexed for 1 min, and 15 μl of the material was pipetted onto an ANDE swab for Rapid DNA Identification. For older bone samples, 100–500mg was processed as above but, following addition of Bone Solution, was subjected to overnight incubation at 56 °C.

Tooth: The cleaning protocol is similar to that of the bone above. If adhering tissues were present, these were further removed by using a sterile scalpel. The crown of the tooth was separated from the lower main body containing the root(s) using a Dremel or by striking with a hammer at an angle toward the top. Tooth fragments were collected using a pair of tweezers or disposable spatula. The root was utilized for analysis.

### Rapid DNA processing

The ANDE Rapid DNA Identification system consists of a fully automated instrument, single-use microfluidic chips, and fully integrated Expert System analysis software. Two types of chips were utilized: I-Chips for Unidentified Human Remains (UHR) samples and A-Chips for Family Reference Sample (FRS) buccal swabs. Both chips perform automated purification, PCR amplification of 27 Short Tandem Repeat (STR) markers (the FlexPlex Assay [18], which includes the 20 loci of the FBI’s CODIS system), electrophoretic separation of amplified and fluorescently labelled fragments, and laser-based fluorescence detection. A major difference between the two chip types is that I-Chips perform a concentration step following DNA purification to maximize the quantity of DNA available for amplification [17, 19, 20].

Up to four swabs are placed into an I-Chip (up to five for an A-Chip), and the chip is placed into the ANDE™ instrument. All required chemical reagents are pre-loaded into the chip, and, following processing, the DNA ID is analyzed automatically by the Expert System, generating DNA IDs in less than 2 h without human intervention or interpretation. The ANDE instrument is ruggedized to Military Standard 810G for transport and use outside of a controlled laboratory environment. Furthermore, the chips are stable at room temperature for six months.

### Conventional laboratory testing

To verify concordance between ANDE® FlexPlex™ chemistry-generated DNA IDs and those generated by conventional laboratory methods, two sample types were shipped to Bode Cellmark Forensics, Inc. (Lorton, Virginia) for testing. Samples presented for concordance testing included bloodcards and sample fragments of skeletal muscle tissue taken at intake. To create a conventional DNA ID that contained all 27 FlexPlex loci, samples were processed using both PowerPlex® Fusion 6C and PowerPlex® 21 (Promega, Madison, WI).

## Results and discussion

FlexPlex27 is a 6-color assay modeled after Promega’s PowerPlex® Fusion 6C with the main difference being the substitution of D6S1043 in place of Penta D. The assay contains all expanded CODIS, UK, Interpol, European Standard, German, and Australian core loci, and D6S1043, an important STR marker broadly used in China [18]. The ANDE 6C instrument, FlexPlex chemistry, consumable chip, and Expert System software received FBI NDIS approval in 2018 [17]. Two types of microfluidic consumables were utilized in this study—the ANDE A-Chip and I-Chip. The A-Chip processes up to five samples in a single run in approximately 94 min [20], and is typically utilized for buccal reference samples [18, 20, 21]. The I-Chip processes up to four samples in approximately 103 min [19] and contains a microfluidic DNA concentration module to enable a lower limit of detection than the A-Chip. Resulting DNA IDs are reported as either full (all loci amplified; 27 for male and 24 for female), partial (when at least one locus did not generate called STR peaks), or background (no alleles called). All blood cards and buccal swabs from intake generated full DNA IDs and were used as references. No discrepancies in the DNA IDs were found between blood cards, buccal swabs, muscle, liver, brain, bone, and tooth, and the DNA IDs from Rapid DNA testing were concordant with results from conventional laboratory processing.

### Buccal samples

Buccal samples are by far the simplest to collect in mass disaster settings and generated consistently good DNA IDs until the onset of maggot infestation. Buccal swabs from exposed bodies generated full or near-full DNA IDs from 2–11 days following placement; failure to generate useful results was entirely due to insect activity. All five samples processed at 3 days generated full DNA IDs as did 4 of 6 samples at 4 days. This finding was confirmed by the three bodies maintained under refrigeration; all time-points tested (up to 3 months) yielded full buccal DNA IDs (Table 3). ESM_1.tiff (under “Electronic Supplementary Material” section) shows representative DNA IDs of fresh buccal samples collected using three different methods for analysis—swabs of oral cavity, swabs of collected tissue lining, and tissue fragments. All methods generated full DNA IDs.

**Table 3:** Summary of buccal sample analyses

Sampling time/donor ID	BDO1	BD02	LD01	LD02	LD03	LD04	LD05	LD06	LD07	LD08
Day 1	27/27	27/27	27/27	24/24	27/27	27/27	24/24	27/27	24/24	27/27
Day 2	27/27	27/27	27/27	24/24	27/27	27/27	24/24	N.D.	N.D.	N.D.
Day 3	27/27	27/27	N.S.	24/24	N.S.	27/27	24/24	N.D.	N.D.	N.D.
Day 4	Background	23/27*	Background*	24/24	N.S.	27/27	24/24	N.D.	N.D.	N.D.
Day 5	Background	26/27*	Background*	24/24	N.S.	27/27	24/24	N.D.	N.D.	N.D.
Day 6	Background	26/27*	Background*	24/24	N.S.	27/27	24/24	N.D.	N.D.	N.D.
Day 7	Background	N.S.	N.S.	20/24*	N.S.	27/27	24/24	27/27	24/24	27/27
Day 8	Background*	N.S.	N.S.	N.S.	N.S.	27/27	23/24	N.D.	N.D.	N.D.
Day 9	Background*	N.S.	N.S.	Background*	N.S.	27/27	18/24	N.D.	N.D.	N.D.
Day 10	N.S.	N.S.	N.S.	N.S.	N.S.	27/27	21/24	N.D.	N.D.	N.D.
Day 11	N.S.	N.S.	N.S.	N.S.	N.S.	27/27*	N.S.	N.D.	N.D.	N.D.
Day 14	N.D.	N.D.	N.D.	N.D.	N.D.	N.D.	N.D.	27/27	24/24	N.D.
1 Month	N.D.	N.D.	N.D.	N.D.	N.D.	N.D.	N.D.	27/27	24/24	27/27
3 Months	N.D.	N.D.	N.D.	N.D.	N.D.	N.D.	N.D.	27/27	N.D.	N.D.

N.D. no sample collection conducted

N.S. no sample available for collection due to infestation or severe tissue deterioration

Buccal samples were processed in A-Chips or I-Chips. Data from I-Chip runs are noted with asterisk (*)

### Muscle

Full DNA IDs were observed from above ground samples from 2–8 days, with 5 of 6 samples tested generating full or good partial DNA IDs by day 6. As expected, the number of called loci decreased with increased exposure time. For example, at day 5, BD02 generated 27 loci, 16 loci were generated at day 6, and no STR peaks were observed by day 7. Some samples could not be collected due to scavenging and tissue disintegration as noted. Overall, biceps (Table 4) and quadriceps (Table 5) generated equivalent results. With refrigeration, all muscle samples generated full DNA IDs for the 3-month duration of the morgue study. ESM_2.tiff shows representative full DNA IDs from day 1 and longer PMIs of both bicep and quadricep tissue samples.

**Table 4:** Summary of biceps analysis. Data presented here were from a consolidated analysis of muscle swabs from intact bodies and swabbed tissue fragments

Sampling time/donor ID	BDO1	BD02	LD01	LD02	LD03	LD04	LD05	LD06	LD07	LD08
Day 1	26/27	27/27	27/27	24/24	27/27	27/27	24/24	27/27	24/24	27/27
Day 2	27/27	27/27	27/27	24/24	27/27	27/27	24/24	N.D.	N.D.	N.D.
Day 3	27/27	27/27	N.D.	24/24	N.D.	27/27	24/24	N.D.	N.D.	N.D.
Day 4	9/27	27/27	Background*	24/24*	N.D.	27/27	24/24	N.D.	N.D.	N.D.
Day 5	27/27*	27/27	Background*	24/24*	Background*	27/27	24/24	N.D.	N.D.	N.D.
Day 6	27/27*	16/27*	Background*	20/24*	N.S.	27/27	24/24*	N.D.	N.D.	N.D.
Day 7	Background*	Background*	N.S.	Background*	N.S.	27/27	24/24*	27/27	24/24	27/27
Day 8	Background*	N.S.	N.S.	Background*	N.S.	27/27	N.S.	N.D.	N.D.	N.D.
Day 9	N.S.	N.S.	N.S.	N.S.	N.S.	27/27*	N.S.	N.D.	N.D.	N.D.
Day 10	N.S.	N.S.	N.S.	N.S.	N.S.	19/27*	N.S.	N.D.	N.D.	N.D.
Day 11	N.S.	N.S.	N.S.	N.S.	N.S.	N.S.	N.S.	N.D.	N.D.	N.D.
Day 14	N.D.	N.D.	N.D.	N.D.	N.D.	N.D.	N.D.	27/27	24/24	27/27
1 Month	N.D.	N.D.	N.D.	N.D.	N.D.	N.D.	N.D.	27/27	24/24	27/27
3 Months	N.D.	N.D.	N.D.	N.D.	N.D.	N.D.	N.D.	27/27	N.D.	N.D.

N.D. no sample collection conducted

N.S. no sample available due to infestation or severe tissue deterioration

Samples processed in I-Chips are noted with asterisk (*)

**Table 5:** Summary of quad muscle analysis. Data presented here were from a consolidated analysis of muscle swabs from intact bodies and swabbed tissue fragments

Sampling time/donor ID	BDO1	BD02	LD01	LD02	LD03	LD04	LD05	LD06	LD07	LD08
Day 1	27/27	27/27	27/27	24/24	27/27	27/27	24/24	27/27	24/24	27/27
Day 2	27/27	27/27	27/27	24/24	27/27	27/27	24/24	N.D.	N.D.	N.D.
Day 3	27/27	27/27	N.D.	24/24	N.D.	27/27	24/24	N.D.	N.D.	N.D.
Day 4	9/27	27/27	Background*	24/24	N.D.	27/27	24/24*	N.D.	N.D.	N.D.
Day 5	Background*	27/27	Background*	24/24	24/24*	27/27	24/24*	N.D.	N.D.	N.D.
Day 6	Background*	Background*	Background*	24/24	N.S.	27/27	N.S.	N.D.	N.D.	N.D.
Day 7	Background*	N.S.	Background*	22/24*	N.S.	27/27	N.S.	27/27	24/24	27/27
Day 8	Background*	N.S.	Background*	N.D.	N.S.	27/27	N.S.	N.D.	N.D.	N.D.
Day 9	N.S.	N.S.	Background*	21/24*	N.S.	16/27*	N.S.	N.D.	N.D.	N.D.
Day 10	N.S.	N.S.	Background*	N.S.	N.S.	18/27*	N.S.	N.D.	N.D.	N.D.
Day 11	N.S.	N.S.	N.S.	N.S.	N.S.	N.S.	N.S.	N.D.	N.D.	N.D.
Day 14	N.D.	N.D.	N.D.	N.D.	N.D.	N.D.	N.D.	27/27	24/24	N.D.
1 Month	N.D.	N.D.	N.D.	N.D.	N.D.	N.D.	N.D.	27/27	24/24	27/27
3 Months	N.D.	N.D.	N.D.	N.D.	N.D.	N.D.	N.D.	27/27	N.D.	N.D.

N.D. no sample collection conducted

N.S. no sample available due to infestation or severe tissue deterioration

Samples processed in I-Chips are noted with asterisk (*)

### Liver

Rapid DNA processing of swabbed liver from exposed remains generated a several-fold range of average signal for a given time point. This is likely due to variability in tissue decomposition, and most of the longer PMI samples were liquefied (Table 6). Samples at 2–3 days PMI generated full DNA IDs as did all samples collected from bodies stored in the morgue under refrigeration. ESM_3.tiff shows full and partial DNA IDs from liver swabs. The 2-day DNA ID is well balanced, but the 4-day DNA ID shows significant sloping, typical of samples with significant degradation of genomic DNA.

**Table 6:** Summary of liver analysis

Sampling time/donor ID	LD01	LD02	LD03	LD04	LD05	LD06	LD07	LD08
Day 1	27/27	24/24	27/27	27/27	24/24	27/27	24/24	27/27
Day 2	27/27	24/24	27/27	27/27	24/24	N.D.	N.D.	N.D.
Day 3	N.S.	24/24	N.S.	27/27	24/24	N.D.	N.D.	N.D.
Day 4	17/27*	24/24	N.S.	N.S.	24/24	N.D.	N.D.	N.D.
Day 5	Background*	24/24	N.S.	N.S.	24/24	N.D.	N.D.	N.D.
Day 6	N.S.	20/24*	N.S.	N.S.	N.S.	N.D.	N.D.	N.D.
Day 7	N.S.	N.S.	N.S.	N.S.	N.S.	27/27	24/24	27/27
Day 14	N.D.	N.D.	N.D.	N.D.	N.D.	27/27	24/24	27/27
1 Month	N.D.	N.D.	N.D.	N.D.	N.D.	27/27	24/24	27/27

N.D. no sample collection conducted

N.S. no sample available due to infestation or severe tissue deterioration

Samples processed in I-Chips were noted with asterisk (*)

### Brain

Brain splatter swabs and calotte fragments were collected for 2 donors, and DNA IDs were generated for all samples at day 3 and for one of four samples by day 7 (Table 7).

**Table 7:** Summary of brain splatter and calotte analysis. Data presented here were from brain splatter swabs and swabbed brain calotte tissue fragments

Sampling time/donor ID	BD01-splatter	BD01-calotte	BD02-splatter	BD02-calotte
Day 1	24/27	27/27	27/27	27/27
Day 2	27/27	10/27	27/27	25/27*
Day 3	21/27*	27/27*	15/27	24/27*
Day 4	Background*	15/27*	27/27*	Background*
Day 5	Background*	Background*	19/27*	Background*
Day 6	Background*	N.S.	24/27*	Background*
Day 7	N.S.	N.S.	26/27*	N.S.

N.S. no sample available due to infestation or severe tissue deterioration

Samples processed in I-Chips were noted with asterisk (*)

### Bone and tooth

Eighty-three of 87 bone samples generated useful DNA IDs, from day 1 through 1 year, with the femur, phalanx, and rib generating full DNA IDs; three had microfluidic chip failures (M.F.) and one gave no called alleles/background ID (Table 8). Overall, the phalanx yielded the best DNA typing success and required relatively small quantities of material (typically 25–100 mg) to generate full DNA IDs for the longer PMIs. Fully desiccated rib material (approximately 6 months and longer) was extremely fibrous and difficult to process with a very light hollow core marrow interior. More surface area of the material was necessary to provide optimal input and generate DNA IDs.

**Table 8:** Summary of bone analysis. Results presented below for all bone types (femur, phalanx, and rib) were processed in an I-Chip

Sampling time/donor ID	BD01	BD02	LD01	LD02	LD03	LD04	LD05	LD06	LD07	LD08
Femur										
Day 1	27/27	27/27	27/27	24/24	27/27	27/27	24/24	N.D.	N.D.	N.D.
Day 30	23/27	27/27	N.D.	N.D.	N.D.	N.D.	N.D.	N.D.	N.D.	N.D.
3 months	N.D.	N.D.	19/27	17/24	15/27	N.D.	24/24	27/27	24/24	20/27
6 months	25/27	23/27	24/27	19/24	25/27	16/27	24/24	22/27	23/24	18/27
12 months	27/27	27/27	27/27	9/24	15/27	26/27	21/24	8/27	14/24	Background
Phalanx										
Day 1	27/27	27/27	27/27	24/24	27/27	27/27	24/24	N.D.	N.D.	N.D.
Day 30	27/27	27/27	N.D.	N.D.	N.D.	N.D.	N.D.	N.D.	N.D.	N.D.
3 months	N.D.	N.D.	27/27	24/24	22/27	N.D.	24/24	27/27	24/24	27/27
6 months	20/27	27/27	27/27	M.F.	22/27	27/27	24/24	M.F.	24/24	27/27
12 months	27/27	N.D.	N.D.	M.F.	N.D.	N.S.	N.S.	26/27	24/24	25/27
Rib										
3 months	N.D.	N.D.	N.D.	N.D.	N.D.	N.D.	24/24	27/27	24/24	27/27
6 months	N.D.	24/27	N.D.	N.D.	N.D.	25/27	24/24	26/27	23/24	26/27
12 months	11/27	27/27	26/27	26/27	26/27	26/27	24/24	13/27	21/24	15/27

N.D. no collection conducted. Rib was included in the middle of the study and so most of the initial time-points for the early donor subjects were not collected for this bone type

N.S. no sample available due to limited material

M.F. microfluidic failure

Tooth processing was also broadly successful, with 24/24 samples generating useful DNA IDs, regardless of time-point and up to the 1 year endpoint of the study (Table 9).

**Table 9:** Summary of tooth analysis

Sampling time/donor ID	BD01	BD02	LD01	LD02	LD03	LD04	LD05	LD06	LD07	LD08
Day 1	N.S.	27/27	27/27	N.S.	27/27	27/27	24/24	N.D.	N.D.	N.S.
Day 30	N.S.	27/27	N.D.	N.S.	N.D.	N.D.	N.D.	N.D.	N.D.	N.S.
3 months	N.S.	N.D.	21/27	N.S.	26/27	N.D.	24/24	27/27	24/24	N.S.
6 months	N.S.	22/27	27/27	N.S.	19/27	27/27	24/24	27/27	24/24	N.S.
12 months	N.S.	7/27	27/27	N.S.	N.D.	24/27	24/24	27/27	24/24	N.S.

N.D. no sample collection conducted per initial planning. Month 12 is the end point of this study

N.S. no sample available. BD01, LD02, and LD08 were edentulous

**Fig. 1:**
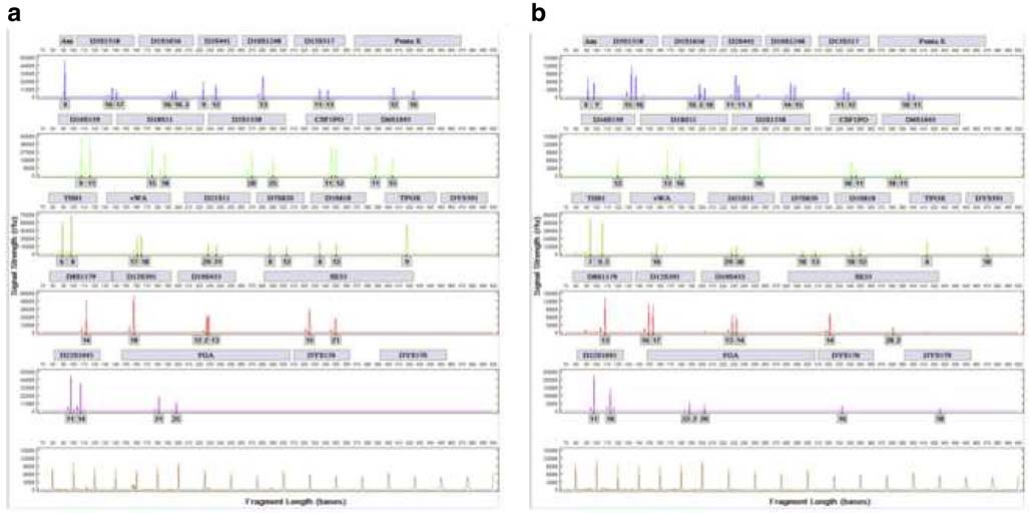
Full DNA IDs from LD05 1-day tooth (**a**) with 1-min pre-processing and BD02 12-month femur (**b**) from an overnight incubation

Figure 1 a shows a representative full DNA ID from tooth at day 1 and Fig. 1b shows a representative full DNA ID from femur at month 12. ESM_4.tiff shows representative full DNA IDs generated from both femur and phalanx from an identical body at month 12 PMI. ESM_5.tiff shows a representative full DNA ID generated from a tooth at month 12 PMI.

Taken together, these data demonstrate the successful application of Rapid DNA Identification of human remains exposed above ground for up to 1 year. In addition to the accuracy and concordance data presented here, precision, resolution, peak height ratio, sensitivity, species specificity, and all other relevant measures meet or exceed required metrics. Table 10 summarizes the overall findings of this study based on the ability of a given sample type to generate a useful DNA ID as a function of time exposure across subjects placed above ground after intake. In the current study, of all data points that generated DNA IDs, the mean number of called loci was 24.63 and the median 26, both sufficiently high to generate low random match probabilities and to be effective in establishing kinship for primary and secondary relationships.

**Table 10:** Summary of findings for tissue types obtained from exposed bodies and ability to generate STR data for identification

Sample-tissue type	Youngest sample without useful data	Oldest sample with useful data	Median of the latest day that gives good sample and useful data
Buccal	Day 3	Day 11	Day 6
Arm muscle	Day 4	Day 10	Day 6
Quad muscle	Day 4	Day 10	Day 5
Liver	Day 3	Day 6	Day 4
Brain	Day 4	Day 7	Day 5.5
Bone-femur	-	Month 12 (at least)	Month 12
Bone-phalanx	-	Month 12 (at least)	Month 12
Bone-rib	-	Month 12 (at least)	Month 12
Tooth	-	Month 12 (at least)	Month 12

The practical application of this data is to allow a first responder to determine which sample type should be collected when one or many sets of exposed human remains are encountered. This determination is based on two related criteria: first, the sample types that are most likely to generate useful DNA IDs using the ANDE system and second, the sample types that are easiest to collect. In the early days following a mass disaster or when a newly deceased body is found, it is clear that buccal swabs are the Rapid DNA sample of choice, and these should be obtained for as long as possible (typically several days of exposure until insect infestation occurs, or months in a refrigerated morgue). If buccal swabs cannot be collected for these or other reasons (e.g., decapitated remains), muscle swabs are reasonable to collect for up to 5 days of exposure. Beyond this time point, the tooth and bone are by far the sample types of choice.

When generating DNA IDs from mass disaster, forensic casework, and mass graves, it is imperative to assess each set of remains to determine the optimal types of samples to process [5, 22]. Temperature, moisture, and oxygen levels in the depositional environment, soil composition, and pH affect the ability to obtain analyzable DNA from biological specimens [23]. When severe degradation and prolonged aging are present, DNA contained within the bone and teeth is protected [24–30] and typically persists for much longer than in soft tissues. The type of bone being analyzed also plays a role in DNA typing success, with denser and more compact bones yielding more DNA than non-weight-bearing structures [31, 32]. The results of the present study are consistent with and expand upon this previous work by demonstrating that these principles can be applied to generating DNA IDs in the field using Rapid DNA Identification technology. Finally, the research presented here with respect to timing of sample type selection and methods for sample preparation may also be useful whether the samples are processed by Rapid DNA Identification or by conventional laboratory analysis.

## Conclusion

The value of Rapid DNA Identification is that the process leads to accurate DNA IDs in a fraction of the time required for conventional laboratory processing. For buccal and tissue swabs, results are generated in less than 2 h, for fresh bone, results are generated in 3 h, and for older bone samples, results are generated overnight. The ability to perform the processing at or near the site of the remains further reduces time to result. In various exercises sponsored by the US National Guard and US Department of Homeland Security, the ANDE system was operated by first responders in tents placed proximal to the mock disaster sites *(*manuscript submitted). In the 2018 California Wildfires, the system was placed at a nearby morgue.

The advent of Rapid DNA technology has changed how DNA can be used to identify human remains following a mass fatality incident. Rapid DNA can be used to generate DNA IDs from human remains in as little as 2 h. The system replaces manual sample manipulations and data interpretation typically performed by highly skilled and qualified scientific personnel in a traditional laboratory. The advantages of Rapid DNA Analysis in field-forward settings are significant and include the following: (1) samples can be obtained and analyzed at the site, eliminating transport time from site to lab and maximizing the opportunity to generate results before remains become significantly degraded; (2) nontechnical users can generate STR results, dramatically expanding the number of samples that can be processed in a given time; and (3) results become available in under 2 h with simplified and easy to use data management software to enable real-time database searching for identity and kinship matches, expediting identification of the deceased and family reunification.

Having established that Rapid DNA is effective for identification of exposed and refrigerated bodies, next steps would be to expand our knowledge to include buried and degraded remains and remains that are much older than 12 months. Preliminary work suggests that these scenarios are amenable to Rapid DNA Identification, but further studies will enable sample collection guidance to be provided to first responders. With data from such studies in hand, it is possible that Rapid DNA will become the method of choice for the identification of human remains.

## Electronic supplementary material

ESM 1 Full DNA IDs from buccal sample of BD01 and analyzed in A-Chip. Day 1 swab of oral cavity and collected at FAC (A), Day 1 scored buccal lining tissue and directly placed in swab chamber (B), and Day 1 swabbed of buccal lining fragment (C). (PNG 1037 kb)

High Resolution Image (TIFF 131495 kb)

ESM 2 Full DNA IDs from muscle tissues. LD02 bicep Day 1 (A) and Day 5 (B) swabbed tissue fragment; LD04 quad Day 1 (C) and Day 8 (D) swabbed tissue fragment. (PNG 1318 kb)

High Resolution Image (TIFF 131495 kb)

ESM 3 DNA IDs from LD01 liver tissue swab at PMI 2 days (A, full DNA ID in A-Chip) and PMI 4 days (B, partial DNA ID in I-Chip). Seventeen of 27 loci amplified and were concordant; D2S441 had Allele 14 drop-out; D13S317, PentaE, D6S1043, D7S820, D5S818, DYS391, DYS576, and DYS570 did not amplify; and SE33 was flagged, indicating that the Expert System did not call this locus. (PNG 647 kb)

High Resolution Image (TIFF 131495 kb)

ESM 4 Full DNA IDs from BD01, 12-month femur (A) and 12-month phalanx (B). (PNG 792 kb)

High Resolution Image (TIFF 131495 kb)

ESM 5 Full DNA ID from LD05 12-month tooth. (PNG 528 kb)

High Resolution Image (TIFF 131495 kb)
