# Supplementary figures and images for: Identification of human remains using Rapid DNA analysis
Source: Int J Legal Med. 2019 Nov 28;134(3):863–72. doi: 10.1007/s00414-019-02186-y (PMC7181457; doi:10.1007/s00414-019-02186-y)

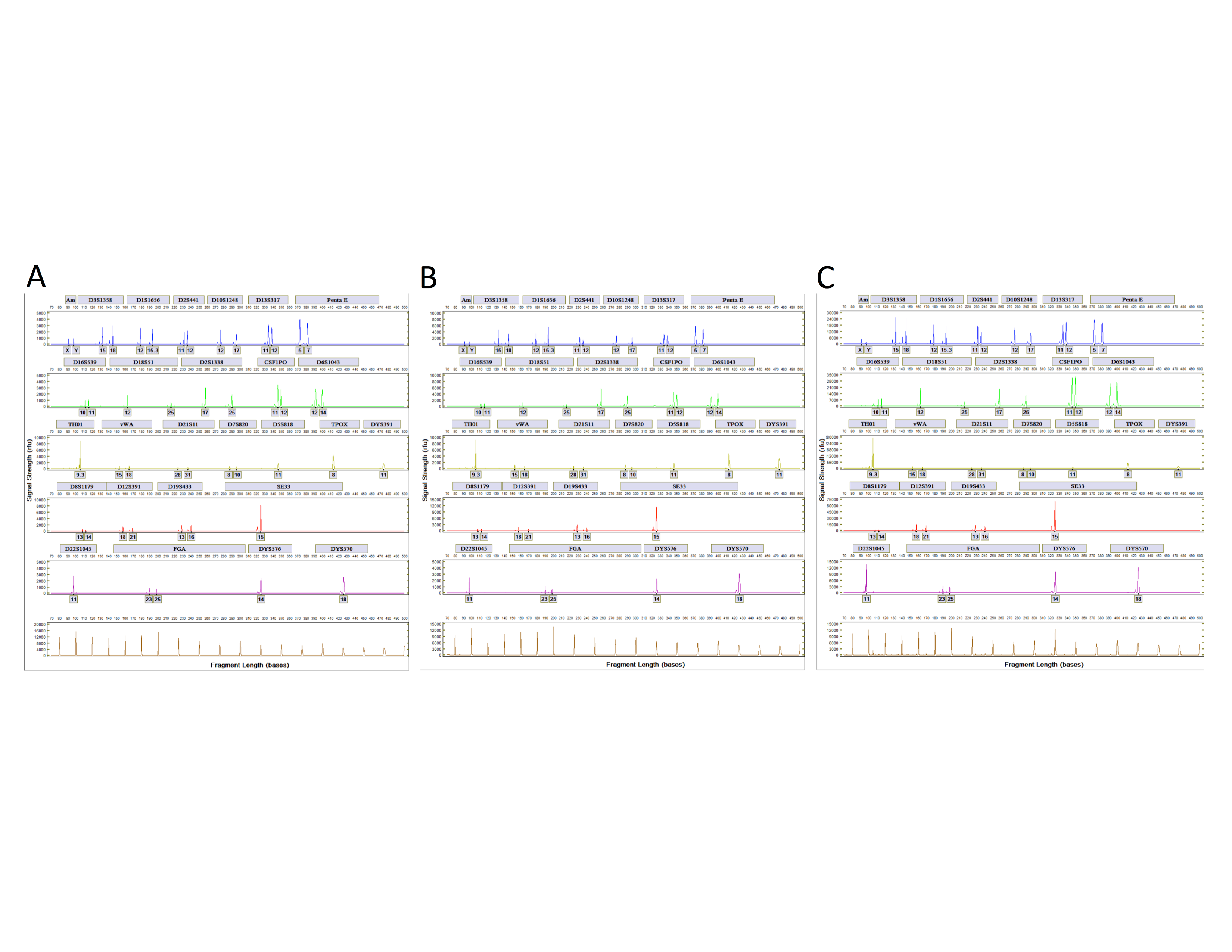

Supplement: Supplementary file 1 — Full DNA IDs from buccal sample of BD01 and analyzed in A-Chip. Day 1 swab of oral cavity and collected at FAC (A), Day 1 scored buccal lining tissue and directly placed in swab chamber (B), and Day 1 swabbed of buccal lining fragment (C). (PNG 1037 kb) [file 414_2019_2186_Fig2_ESM.png]

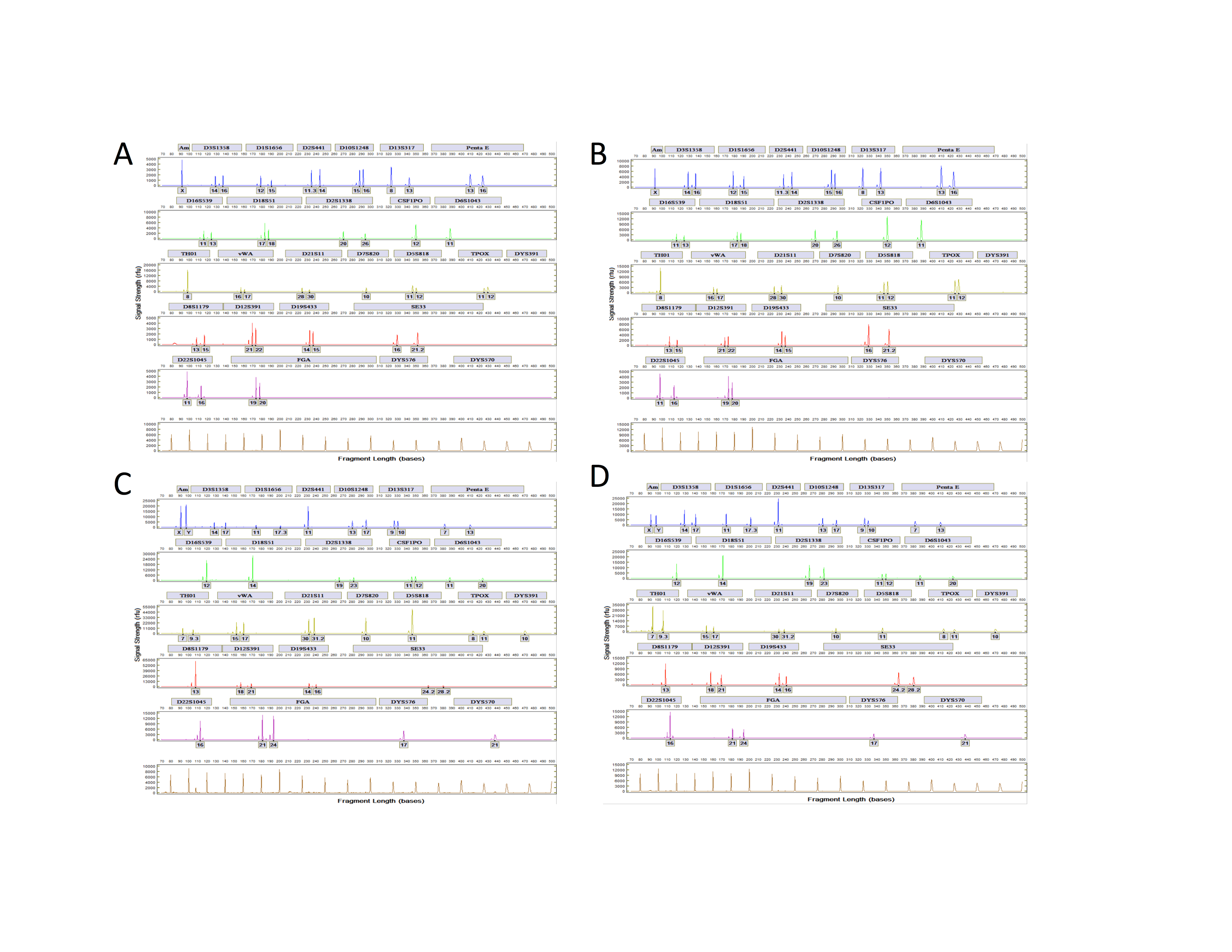

Supplement: Supplementary file 3 — Full DNA IDs from muscle tissues. LD02 bicep Day 1 (A) and Day 5 (B) swabbed tissue fragment; LD04 quad Day 1 (C) and Day 8 (D) swabbed tissue fragment. (PNG 1318 kb) [file 414_2019_2186_Fig3_ESM.png]

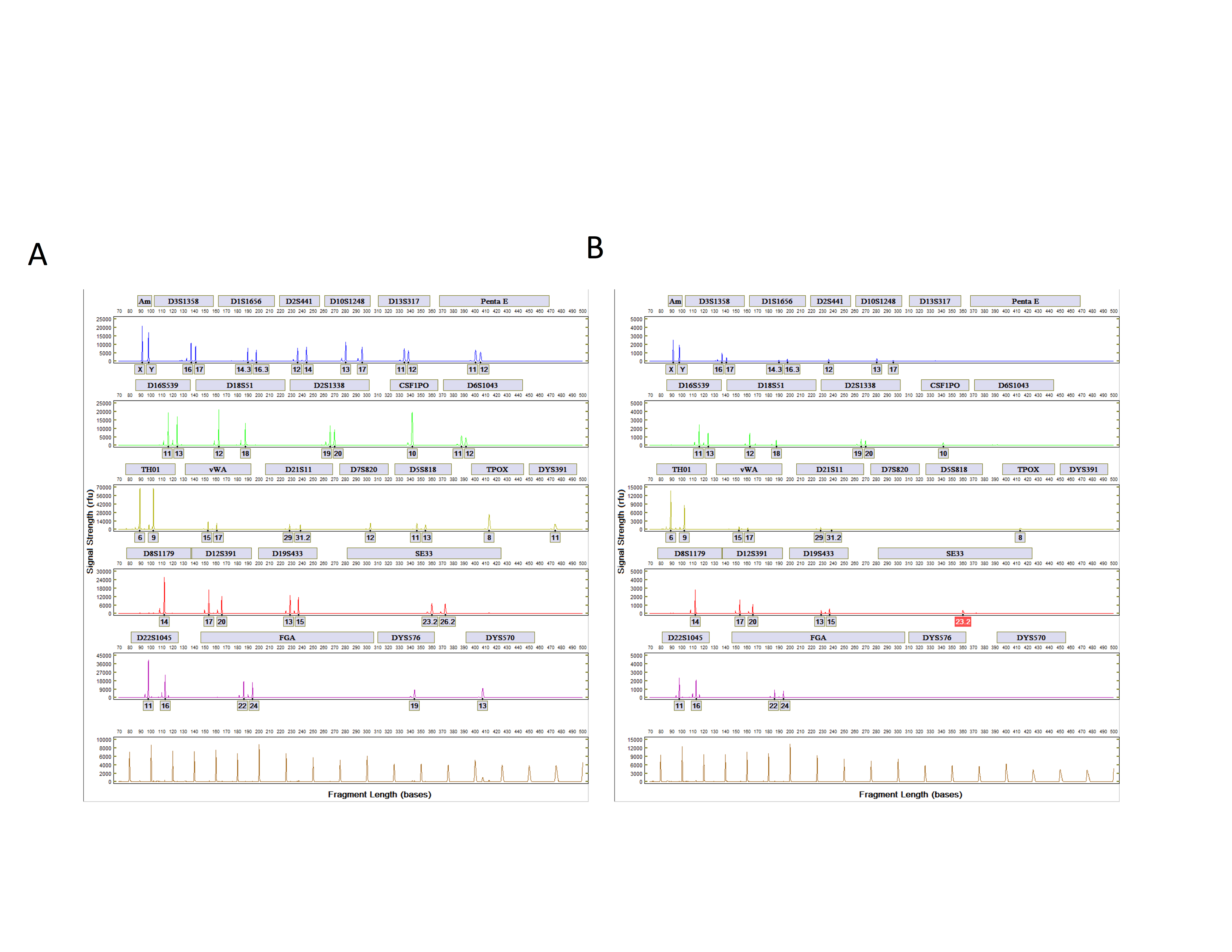

Supplement: Supplementary file 5 — DNA IDs from LD01 liver tissue swab at PMI 2 days (A, full DNA ID in A-Chip) and PMI 4 days (B, partial DNA ID in I-Chip). Seventeen of 27 loci amplified and were concordant; D2S441 had Allele 14 drop-out; D13S317, PentaE, D6S1043, D7S820, D5S818, DYS391, DYS576, and DYS570 did not amplify; and SE33 was flagged, indicating that the Expert System did not call this locus. (PNG 647 kb) [file 414_2019_2186_Fig4_ESM.png]

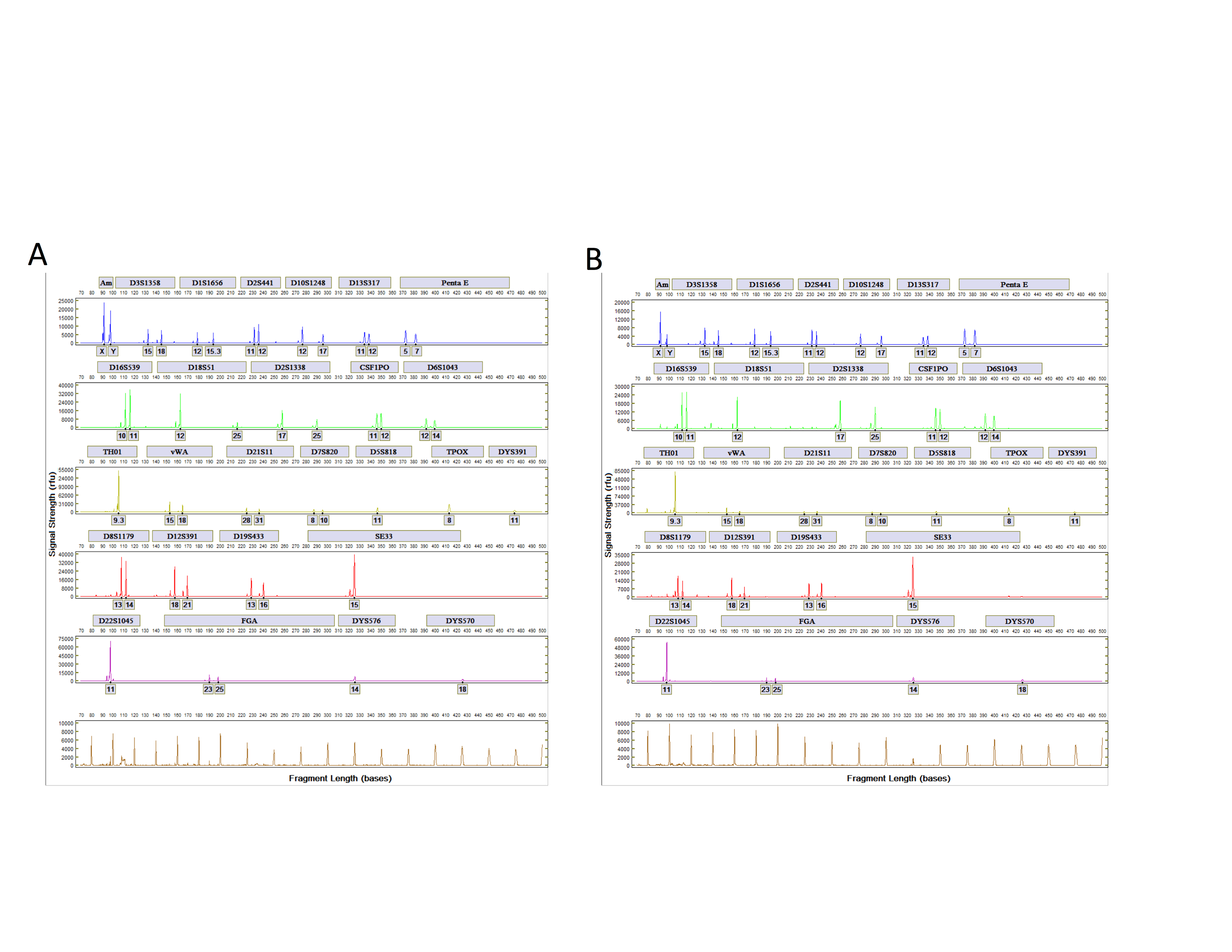

Supplement: Supplementary file 7 — Full DNA IDs from BD01, 12-month femur (A) and 12-month phalanx (B). (PNG 792 kb) [file 414_2019_2186_Fig5_ESM.png]

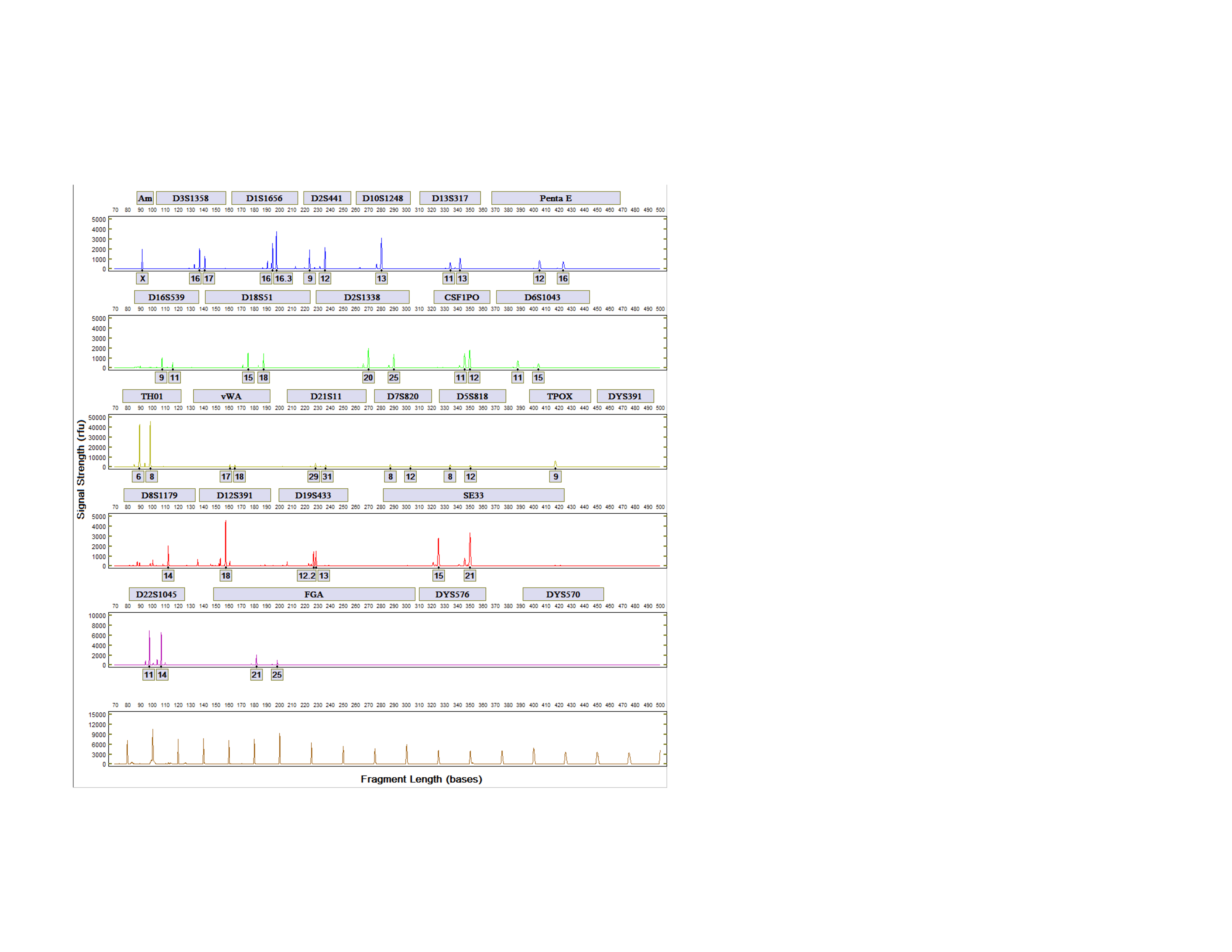

Supplement: Supplementary file 9 — Full DNA ID from LD05 12-month tooth. (PNG 528 kb) [file 414_2019_2186_Fig6_ESM.png]
